# Modelling the Association between Core and Discretionary Energy Intake in Adults with and without Obesity

**DOI:** 10.3390/nu11030683

**Published:** 2019-03-22

**Authors:** Mackenzie Fong, Ang Li, Andrew J Hill, Michelle Cunich, Michael R Skilton, Claire D Madigan, Ian D Caterson

**Affiliations:** 1 The Boden Institute of Obesity, Nutrition, Exercise & Eating Disorders, Charles Perkins Centre, University of Sydney, Sydney, NSW 2006, Australia; a.li@sydney.edu.au (A.L.); A.J.Hill@leeds.ac.uk (A.J.H.); michelle.cunich@sydney.edu.au (M.C.); michael.skilton@sydney.edu.au (M.R.S.); claire.madigan@phc.ox.ac.uk (C.D.M.); ian.caterson@sydney.edu.au (I.D.C.); 2 Sydney Health Economics, Sydney Local Health District, Sydney, NSW 2050, Australia; 3 Division of Psychological & Social Medicine, Institute of Health Sciences, University of Leeds, Leeds LS2 9NL, UK; 4 Nuffield Department of Primary Care Health Sciences, University of Oxford, Oxfordshire OX2 6GG, UK

**Keywords:** discretionary intake, core intake, dietary compensation, beverage consumption, weight control, obesity

## Abstract

Background: Many dietary recommendations for weight control rely on the assumption that greater core food intake will displace intake of energy-dense discretionary foods and beverages. However, there is little evidence to support these assumptions. This study examined the naturalistic relationship between daily core and discretionary energy intake, and with discretionary food and discretionary beverage intake, separately. The impact of weight status on these associations was also examined. Method: One hundred participants completed a four-day (non-consecutive) estimated food diary. Discretionary foods and beverages were identified by reference to the Australian Dietary Guidelines. Non-discretionary items were considered core items. Simultaneous-equation random effects models using disaggregated dietary data controlling for sociodemographic variables were used to determine the association between various dietary components. Result: Core energy intake correlated negatively with discretionary energy intake (cross-equation correlation, ρ = −0.49 (95% CI: −0.57, −0.39)). Its correlation with discretionary foods (−0.47 (−0.56, −0.37)) was stronger than that with discretionary beverages (−0.19 (−0.30, −0.07)) The correlation between core energy intake and discretionary energy intake was significantly stronger in participants who did not have obesity (−0.67 (−0.71, −0.50)) than those with obesity (−0.32 (−0.46, −0.17)) (*p* = 0.0002). Conclusions: Core and discretionary energy intake share an inverse and potentially bidirectional, relationship that appears to be stronger with discretionary foods than discretionary beverages. These relationships were significantly weaker in participants with obesity which may indicate less precise dietary compensation in these individuals. While strategies that promote greater intake of core foods may assist with weight maintenance in individuals of healthy weight, its impact in individuals with obesity may be limited. These strategies should be accompanied by direct messages to reduce commensurately the intake of discretionary items, with special attention paid to discretionary beverage consumption.

## 1. Background 

Energy-dense, nutrient-poor discretionary foods and beverages are a major component of Westernised food landscapes. Among the most popular items are cakes, confectionery, cereal/nut/fruit/seed bars, crisps, biscuits, alcoholic beverages and sugar sweetened beverages (SSBs) [1,2]. Within the context of a nutritious diet and adequate levels of physical activity, a small amount of discretionary energy can add variety and enjoyment to eating [3]. However, discretionary items are relatively cheap, promoted aggressively and available readily, and, as such, are consumed rarely within recommended levels. Due to their high sugar and/or fat content, discretionary items are inherently energy-dense, making them an important contributor to inadvertent overeating or “passive overconsumption” [4]. Around 35% of total energy intake (TEI) of adults in Australia [5] and the US [6,7] comes from discretionary items—US guidelines recommend a limit of 13% [8]. Excessive discretionary energy intake (DEI) has several implications. Perhaps of most concern is the positive association with TEI, subsequent weight gain, and risk of obesity. Nationally representative data for American adults have been used to demonstrate that those with the highest DEI have significantly greater TEI than those with lower DEI [9]. Substantive epidemiological research shows a positive association between DEI and BMI [2,10], and, accordingly, the World Health Organisation highlights the importance of reducing DEI to quell rates of obesity [11].

Rather than emphasising explicitly the restriction of DEI for weight control, clinical practice guidelines [12] and public health messages [13] tend to promote greater intake of non-discretionary core foods. Core foods include cereals and grains, lean meat and plant-based proteins, low fat dairy products, fruits and vegetables. These foods have relatively low energy-density, are satiating [14,15], and fruit and vegetable intake in particular has been shown to be associated negatively with DEI [2,10]. However, findings of RCTs promoting greater fruit and vegetable intake for weight loss do not support its efficacy in adults [16,17,18] or children [19] with overweight or obesity, a finding confirmed in a recent systematic review and meta-analysis [20]. Therefore, to improve the effectiveness of dietary recommendations for weight management, an examination of the underlying assumptions, and a clearer understanding of the relationship between DEI and intake of core foods is needed. 

Importantly, these recommendations are predicated on the assumption that core and discretionary intakes share an inverse relationship, connoting dietary compensation, i.e., eating more core foods will be accompanied by a compensatory reduction in discretionary intake, thereby maintaining TEI and promoting weight stability and potentially weight loss [21,22]. However, empirical evidence to support this assumption is sparse and limited in its examination of dietary composition. Field studies in adults conducted over several days observe compensation in overall energy intake [23,24], yet dietary composition is not assessed. Houchins and colleagues [25] observed that, when provided with fruits and vegetables surplus to their habitual diet, participants of healthy weight compensated for this additional energy and their weight remained stable. However, participants with obesity exhibited weaker dietary compensation, which precipitated as significant weight gain over eight weeks. Interestingly, in one field study, the authors observed that TEI was greater on days that participants consumed SSBs, indicative of poorer dietary compensation induced by beverage consumption [23]. This is concordant with growing evidence purporting weaker satiety and appetitive responses following consumption of SSBs and other nutritive beverages compared to nutritionally-matched solid foods [25,26,27,28]. As such, energy obtained from discretionary beverages may be a significant driver of total energy intake, as suggested by findings of several studies showing a stronger relationship between discretionary beverages and BMI, than with discretionary foods [10,29]. Therefore, examining separately the relationships of discretionary foods and discretionary beverages with core energy intake (CEI) across BMI categories is warranted.

To develop a more accurate understanding of the relationship between core and discretionary intake with real-life applicability, several factors need to be considered. First, the relationship between core and discretionary intake may be bidirectional. In previous work examining the association of fruit and vegetable intake and DEI [2,10], DEI is modelled as the dependent variable and fruit and vegetable intake is included as an explanatory variable. This specification implies a unidirectional association, i.e., fruit and vegetable intake affects DEI. However, it is plausible that this relationship also occurs in the opposite direction, i.e., DEI may also affect fruit and vegetable intake [9]. Second, several sociodemographic factors have been associated consistently with both core [2,30] and discretionary intake [2,9,10]. Therefore, analyses that account for these sociodemographic factors (such as age, sex, socioeconomic status, education) in both dietary components are needed. Third, while fruit and vegetable intake and weight control has been studied widely, to the best of the authors’ knowledge, research examining overall core food intake is virtually absent despite evidence showing that foods such as nuts [31,32], legumes [10], wholegrains [33] and low-fat dairy products [2] are also associated with weight control. Studying total CEI may reflect a more flexible and real-world approach than studying fruit and vegetable intake exclusively, especially given that several barriers may prevent the adoption of their increased consumption, e.g., taste aversion [34,35,36] and perceived greater cost [37]. 

To address existing knowledge gaps, the primary aim of the current study was to determine the intraindividual associations between daily CEI and DEI, and to determine those between CEI and daily discretionary foods and discretionary beverage, separately. The secondary aim was to determine the impact of weight status on the relationship between these dietary variables. We hypothesised that CEI would correlate negatively with DEI and that this correlation would be stronger with discretionary foods than discretionary beverages. It was also hypothesised that these correlations would be weaker in participants with obesity. Simultaneous-equation models were used to control for multiple variables and to account for the potential bidirectionality of the associations between dietary components. 

## 2. Methods 

### 2.1. Study Participants 

One hundred adults were recruited, 50 of healthy weight (BMI 18.5–24.9 kg/m^2^) and 50 with obesity (BMI ≥ 30.0 kg/m^2^). Participants were recruited through a post on the University of Sydney research volunteer website, flyers posted around the University campus and advertisements emailed to registrants of the Boden Institute clinical trials database. The advertisements invited individuals to participate in a study investigating a broad range of eating behaviours and their relationship to weight control. The advertisement did not state explicitly the authors’ intention to examine discretionary intake to reduce the risk of social desirability bias and subsequent underreporting. Participants provided informed written consent prior to study enrolment. To be eligible to participate, participants needed to be aged ≥18 years and able to complete the study materials adequately. Participants were excluded from the study if they: were currently enrolled in a weight management program, were on a restrictive diet, had gained or lost 5% of their body weight in the previous three months, were shift workers, were currently pregnant or breast feeding, had an eating disorder, had previous bariatric surgery, or were currently/previously enrolled in a nutrition degree. 

### 2.2. Anthropometry

Height was measured to the nearest centimetre using a wall mounted stadiometer. Weight was measured to the nearest 0.1 kg using calibrated, digital scales. BMI was calculated in kg/m^2^. Measures were collected with participants in light clothing and shoes removed. Waist circumference was measured at the mid-point between the highest point of the iliac crest and lowest part of the costal margin in the midaxillary line. Measurements were record to the nearest 0.5 cm. 

### 2.3. Background Questionnaires

Participants completed a questionnaire at Visit 1 that collected demographic information including age, sex, education level, health status and postcode. The latter was used to determine participants’ socioeconomic indexes for areas (SIEFA) decile, which provided a broad measure of socio-economic status [38]. 

### 2.4. Dietary Intake

Participants completed a non-consecutive, four-day estimated food diary comprising three weekdays and one weekend day. At Visit 1, the study dietitian educated participants on how to record intake as accurately as possible and encouraged participants to maintain their usual diet during the recoding period. Participants recorded the time of consumption, the food/beverage description (type, varietal specifications, e.g., low-fat, product name, and cooking method) and estimated portion-size using weights, volumes, dimensions (height × width × depth) and household measures. At Visit 2, the study dietitian assessed the food diary for completeness and prompted participants for clarification or additional information where required. 

### 2.5. Nutrient Analysis and Coding

Dietary data were analysed using Xyris Foodworks Nutrition Analysis software (version 9, High Gate Hill, Australia) [39]. TEI was extracted from analyses and all energy-yielding foods and beverages were categorised as either DEI or CEI. Food and beverages that contributed to DEI were identified by the study dietitian with reference to the Australian Dietary Guidelines [3] and methods used in previous studies [40,41]. CEI were foods and beverages that did not meet criteria for discretionary items and comprised several food groups including low-fat dairy products and alternatives, legumes, fruits, vegetables, lean meat, grains and cereals. All items that contributed to DEI were categorised as either foods or beverages. Semi-solid discretionary items such as high fat yoghurts and soups were classified as foods given that these items have been shown to elicit high satiety [42] and are usually eaten as foods with cutlery as opposed to consumed as a drink. For the purposes of this research, full fat milk added to cereal was not considered a discretionary beverage. Drinks with dissolved nutrients, e.g., sugar added to teas and coffees, cordials and flavouring powders, were considered discretionary in line with the Beverage Guidance Panel recommendations [43]. 

### 2.6. Validity of Dietary Intake

Reported energy intake was assessed for validity using the Goldberg method [44], which involves calculating the ratio between reported TEI and BMR based on the Harris Benedict equation [45]. A ratio of less than 0.9 indicates that reported TEI is not consistent with energy intake required for a normal (non-bedbound) lifestyle. Participants whose reported energy intake yielded TEI:BMR <0.9 were considered under reporters and their data excluded from analyses. 

### 2.7. Study Procedures

Anthropometric measurements were collected, baseline questionnaires were administered, and a four-day estimate food diary was dispensed at Visit 1. The study materials were returned to the researcher approximately ten days later at Visit 2. Participants received a $30 voucher recompense. The study was approved by the Sydney Local Health District Human Research Ethics Committee (Protocol Number X17-0228).

### 2.8. Statistical Analysis

Dietary data were disaggregated to assess the individual-level, daily relationship of dietary components. Multi-equation multi-level models were used to estimate the relationship between dietary components. Specifically, TEI (kJ), DEI (kJ), CEI (kJ), %TEI from DEI, %TEI from CEI, discretionary beverages (kJ) and discretionary foods (kJ) were modelled using simultaneous-equation random effects models, with random effects specified at the individual level [46]. All the specifications controlled for sex, age (<35 years, 35–64 years, or ≥65 years), education (completed post high school education or not), obesity status, day of the week energy intake was reported (weekend or weekday) and socioeconomic status (SEIFA top quintile (highest level of socioeconomic status)) or below). Estimated results from the simultaneous-equation random effects models are presented as the observation-level cross-equation correlation, ρ (95% Confidence Intervals (CI)). All tests of significance of the explanatory variables were conducted at the significance level of 0.05. Analyses were performed using Stata software version 14.0 [47]. 

**Figure 1:**
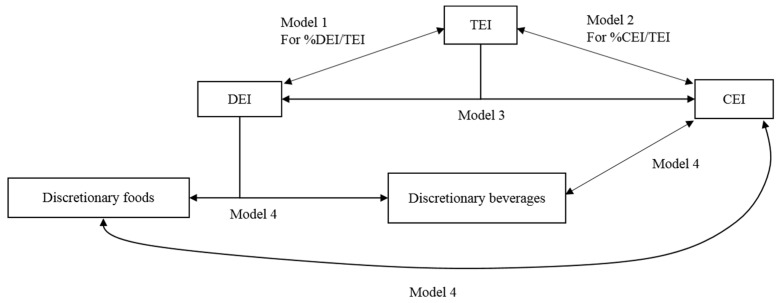
The relationship between various dietary components assessed in the current study using simultaneous-equation random effects models. TEI, total energy intake; DEI, discretionary energy intake; CEI, core energy intake.

A visual representation of the relationships between various dietary components and the modelling strategies that were studied is presented in Figure 1. Four multi-equation models were estimated: equations of TEI and proportion of TEI from DEI (%DEI/TEI) (Model 1), equations of TEI and proportion of TEI from CEI (%CEI/TEI) (Model 2), equations of DEI and CEI (Model 3), and equations of discretionary foods, discretionary beverages, and CEI (Model 4). 

## 3. Results 

The reported TEI of seven participants yielded a Goldberg ratio of <0.9. These participants were excluded and the data for 93 participants formed the analysis sample. Most participants were female (79), their mean age was 45.7 years (SD = 21.0), and 47 had obesity. Participants provided a total of 368 daily food diary entries. The sample mean TEI was 8477 kJ/day (SD = 1893). Discretionary intake was 39.8% (SD = 16.8) of TEI, of which a mean of 12.0% (SD = 13.9) was from beverages.

### 3.1. Relationship between Sociodemographic Variables and DEI and CEI

The estimates from simultaneous-equation random effects model of CEI and DEI with the sociodemographic factors included as covariates are shown in Table 1. In the combined sample, participants aged 35–64 years (−1246 kJ (−2355, −136)) or ≥65 years (−2281 kJ (−3494, −1068)) consumed less DEI than those aged <35 years, holding all other variables constant. Participants with obesity consumed more DEI (1313 kJ (253, 2373)) and TEI recorded on a weekend day was greater than TEI recorded on a weekday (650 kJ (118, 1182)). Males consumed more CEI than females (2313 kJ (1430, 3196)), and those in the top SEIFA quintile (representing greater socioeconomic advantage) consumed less CEI (−855 kJ (−1561, −149)). 

### 3.2. Relationship between TEI, DEI, and CEI by Obesity Status 

**Figure 2:**
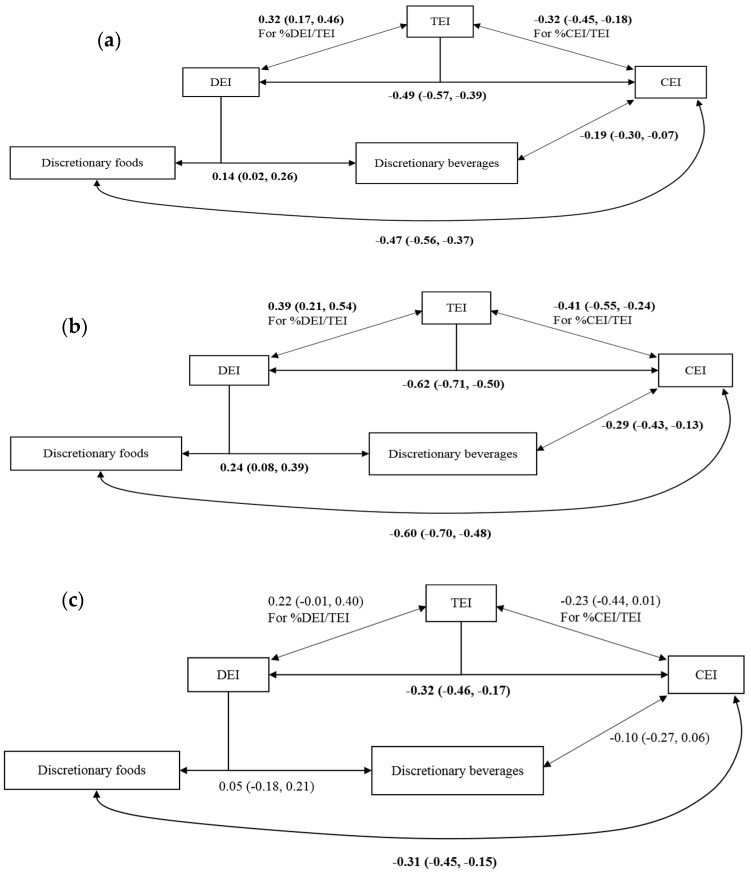
Diagrammatic representations of the correlations among TEI, CEI, DEI, discretionary foods and beverages in: (**a**) the combined participant sample; (**b**) participants without obesity; and (**c**) participants with obesity. Results are presented as cross-equation correlation ρ (95% CI). Models were adjusted for sex (male or female), age (<35 years, 35–64 years, or ≥65 years), education (completed post high school education or not), BMI category (has or does not have obesity), day of the week dietary intake was reported (weekend or weekday) and socioeconomic status (SEIFA top quintile or below). Correlations written in bold face are significant at the 5% level. TEI, total energy intake; DEI, discretionary energy intake; CEI, core energy intake.

The estimated cross-equation correlations between the various dietary components adjusting for sociodemographic factors are shown in Table 2 and are presented diagrammatically in Figure 2a–c. TEI correlated positively with %DEI/TEI (0.32 (0.17, 0.46)) (Model 1) and negatively with %CEI/TEI (−32 (−45, −18)) (Model 2). DEI was negatively correlated with CEI (−0.49 (−0.57, −0.39)). CEI correlated more strongly with discretionary foods (−0.47 (−0.56, −0.37)) than with discretionary beverages (−0.19 (−0.30, −0.07)) (Model 4).

The correlation between DEI and CEI was significantly stronger in participants of healthy weight (−0.62 (−0.71, −0.50)) than those with obesity (−0.32 (−0.46, −0.17)) (*p* = 0.0002). While discretionary beverage intake in both groups was correlated negatively with CEI, this was only statistically significant in participants of healthy weight (−0.29 (−0.43, −0.13)).

## 4. Discussion 

This study examined and modelled the intraindividual relationship between daily CEI and DEI in adults, which, to the best of the authors’ knowledge, has not been undertaken previously in this literature. CEI and DEI were correlated negatively, although this was weaker for energy intake from discretionary beverages. The modest inverse association of CEI with DEI was weaker in participants with obesity. These associations appeared additive, such that, for participants with obesity, energy intake from discretionary beverages did not offset CEI. 

That daily DEI and CEI correlated negatively confirms previous assumptions that these two variables are discrete dietary components that share an inverse relationship in the absence of dietary intervention. A change in the intake of one variable corresponded to a change in the other in the opposite direction. The precise reasons for this observation cannot be determined with certainty, although likely involve homeostatic mechanisms that seek to maintain TEI. While previous naturalistic studies conducted over several days have reported energy compensation [48], research investigating specific food types is lacking. Given the current findings, hypothetically, energy intake from discretionary items may be off-set by a compensatory reduction in energy from core foods so that TEI remains relatively stable, mitigating the increased risk of weight gain. This may explain why some individuals who consume a relatively high proportion of discretionary energy are able to maintain a healthy weight. Dietary compensation has physiological origins involving neuroendocrinological mechanisms that enhance satiety and attenuate further eating in order to maintain energy homeostasis. However, cognitive behavioural influences may also be involved. For instance, Lenne and colleagues observed that over one third of adults surveyed reported pre-emptively adjusting eating behaviour prior to dietary transgressions at a state fair, such as eating less or choosing more healthy foods, referred to as pre-compensation [49]. 

The strength of this relationship was significantly weaker in participants who had obesity. To give real-life context, intake of one dietary component may not be accompanied by a reduction in the other of the same magnitude in these individuals. This suggests that increased intake of core foods may not displace DEI in these individuals due to less stringent and less precise dietary compensation. This is concordant with previous observations whereby additional energy commensurate to the provision of fruits and vegetables was compensated for by participants of healthy weight, but not by those with obesity [25]. Similarly, an empirical feeding study in adolescents found that TEI was significantly greater on fast-food days compared to non-fast-food days in adolescents with overweight. This was not observed in healthy weight counterparts [50], indicating a compensatory reduction in subsequent energy intake in these participants. 

Separating DEI into foods and beverages enabled a more detailed examination of the relationship between DEI and CEI. CEI was correlated more strongly with discretionary foods than with discretionary beverages. In theory, intake of a discretionary beverage may not be accompanied by a reduction in CEI of the same magnitude as that induced by discretionary food. This finding is consistent with a growing body of literature reporting weaker appetitive responses and poorer dietary compensation associated with consumption of beverages compared to solid food, regardless of macronutrient composition [25,26,28,51]. While there is still conjecture surrounding the exact mechanisms that drive this observation, several theories have garnered supportive evidence. Beverages have faster gastric transit times [52,53], demand less oral processing [54,55], reduce ghrelin suppression [27,56] and evoke lower cognitive perception of expected satiety [27,29]. Each of these factors contributes ostensibly to lower post-ingestive satiety or the perception thereof, compared to solid foods. 

Several physiological, psychological and behavioural factors may contribute to the slight “decoupling” of CEI and DEI in participants with obesity. Extensive research shows that satiety responsiveness in individuals with obesity is weaker than in those of a healthy weight [57,58,59]. Moreover, problematic eating behaviours and psychological traits such as disinhibited eating [60], food-related cue reactivity [61], reward from eating [62] and counterregulatory eating following breach of dietary restraint [63] are purportedly heightened and more prevalent in individuals with obesity. In addition, poor energy compensation associated with beverage consumption may be more pronounced in participants with obesity; however, current evidence remains inconclusive [26,28]. Together, these factors may work to decouple DEI from CEI, resulting in dysregulated energy compensation in these individuals. It is also possible that other sociodemographic factors that were unaccounted for in the current study contributed to this difference, e.g., nutrition knowledge, income. 

These findings have practical implications for developing dietary recommendations for weight management at a clinical and public health level. First, they challenge popular advice that prioritises greater core food intake in order to displace DEI for weight loss. The negative correlation between DEI and CEI was relatively weak in participants who had obesity. Increasing intake of core foods without a substantial, commensurate reduction in other dietary components may lead to increased body weight if continued. Strategies that encourage increased core intake combined with specific recommendations to restrict DEI [15] and education on behavioural energy compensation may be more effective for weight loss. Second, the consumption of discretionary beverages warrants special attention given its relatively weak and negative correlation with CEI. While moderation of SSB consumption has been adopted into wider public discourse and policy, this has not been extended to other energy-yielding beverages. This message may be especially pertinent for individuals with obesity who appear to consume a greater proportion of DEI from beverages [10]. 

The empirical methodology used in this study has several strengths. Collecting participants’ dietary intake using four-day estimated food diaries provided rich, quantitative data without compromising its integrity due to respondent fatigue as with seven-day diaries [64]. Investigating total CEI rather than fruit and vegetable intake exclusively provided more real-world applicability, especially given that several barriers may prevent the adoption of greater vegetable intake [35,36,37]. Analysing these data in a disaggregated form, such that each day was a separate observation, facilitated the investigation of over 360 unique observations. This enabled the analysis of the daily, individual-level relationship of dietary components, which, to the best of the authors’ knowledge, has not been done previously. The use of simultaneous-equation models accommodated for the putative bidirectional relationship between dietary components and controlled contemporaneously for sociodemographic factors associated with both DEI and CEI. This statistical approach provided a more real-world and accurate estimate of their relationship beyond single equation regressions.

The findings presented in this study are limited by a few factors. The direction of the relationships between dietary variables cannot be determined within the scope of the study’s design. In addition, most participants were female, limiting the generalisability of findings to the wider population. Further, it is possible that the current observations may be explained by other factors that were not accounted for in the current study. For instance, addictive behaviours, such as smoking, may drive the intake of discretionary foods [65] but was not included as a covariate in analyses. Future research would benefit from accounting for factors such as nutrition education, income and addictive behaviours to reduce the impact of potential confounders. In addition, previous work has shown that corrective dietary responses may occur three to four days following deviations from average energy intake [24]. Accounting for this lag time in future work may provide a better understanding of the temporal relationship between DEI and CEI.

## 5. Conclusions

In summary, DEI and CEI appear to share a natural, inverse, and potentially bidirectional relationship that may be indicative of dietary compensation. The correlation of CEI with discretionary beverages was weaker than its correlation with discretionary foods, suggesting that dietary compensation is less complete on days that beverages are consumed. In general, the relationships between these dietary components were weaker in participants with obesity, which may connote weaker dietary adjustment in these individuals. Our findings suggest that promotion of core food consumption may assist with weight maintenance in participants of healthy weight, but its impact in individuals with obesity may be limited. Increased core food consumption is unlikely to have meaningful effect as a weight loss strategy if it is the only strategy implemented and should be accompanied by direct messages to reduce commensurately the intake of discretionary items, with special attention paid to discretionary beverage consumption. 

## Figures and Tables

**Table 1:** CEI and DEI and associations with sociodemographic variables.

Explanatory Variables	All Participants (*n* = 93)	
	DEI	CEI
Male	21 (−893, 935)	**2313 (1430, 3196)**
35–64 years	**−1246 (−2355, −136)**	929 (−144, 2002)
≥65 years	**−2281 (−3494, −1068)**	519 (−654, 1691)
Completed or undertaking post-high school education	−342 (−1022, 339)	457 (−200, 1115)
Obesity	**1313 (253, 2373)**	−471 (−1496, 555)
Weekend	**650 (118, 1182)**	−161 (−577, 255)
Top SEIFA quintile	153 (−577, 883)	**−855 (−1561, −149)**

Results are from estimated simultaneous equation models with random effects. Results are presented as β coefficient (95% CI), where β represents the difference in energy intake (kJ) compared to the reference group who are female, aged below 35 years, have not completed/currently undertaking post-high school education, are of healthy weight, reporting dietary intake on weekdays and living in a relatively disadvantaged area with SEIFA below top quintile. Coefficients written in bold face are significant at the 5% level. DEI, discretionary energy intake; CEI, core energy intake.

**Table 2:** Correlations between dietary components in the total sample and by obesity status.

Variables Included in Simultaneous Equations	All Participants	Healthy Weight Participants	Participants with Obesity
Model 1			
TEI and %DEI/TEI	**0.32 (0.17, 0.46)**	**0.39 (0.21, 0.54)**	0.22 (−0.01, 0.40)
Model 2			
TEI and %CEI/TEI	**−0.32 (−0.45, −0.18)**	**−0.41 (−0.55, −0.24)**	−0.23 (−0.44, 0.00)
Model 3			
DEI and CEI	**−0.49 (−0.57, −0.39)**	**−0.67 (−0.71, −0.50)**	**−0.32 (−0.46, −0.17)**
Model 4			
Discretionary food and discretionary beverages	**0.14 (0.02, 0.26)**	**0.24 (0.08, 0.39)**	0.05 (−0.18, 0.21)
Discretionary food and CEI	**−0.47 (−0.56, −0.37)**	**−0.60 (−0.70, −0.48)**	**−0.31 (−0.45, −0.15)**
Discretionary beverages and CEI	**−0.19 (−0.30, −0.07)**	**−0.29 (−0.43, −0.13)**	−0.10 (−0.27, 0.06)
Observations	364	183	185

Simultaneous equation random effects models were used to determine the correlations between different dietary variables. Results are presented as cross-equation correlations, ρ (95% CI). Models were adjusted for sex, age (<35 years, 35–64 years, or ≥65 years), education (completed or undertaking post-high school education or not), weight status (for analyses in all participants), day of the week dietary intake was reported (weekend or weekday), and socioeconomic status (SEIFA top quintile or below). Correlations written in bold face are significant at the 5% level. TEI, total energy intake; DEI, discretionary energy intake; CEI, core energy intake.

## Abbreviations

CEI	core energy intake
DEI	discretionary energy intake
SEIFA	socioeconomic indexes for areas
SSB	sugar-sweetened beverage
TEI	total energy intake
